# Greenhouse gas emissions due to unnecessary antibiotic prescriptions

**DOI:** 10.1017/ash.2024.354

**Published:** 2024-09-04

**Authors:** Emily S. Spivak, Jessica Tobin, Adam L. Hersh, Alexis P. Lee

**Affiliations:** 1 Division of Infectious Diseases, Department of Internal Medicine, University of Utah School of Medicine, Salt Lake City, UT, USA; 2 University of Utah School of Medicine, Salt Lake City, UT, USA; 3 Division of Infectious Diseases, Department of Pediatrics, University of Utah School of Medicine, Salt Lake City, UT, USA; 4 Environmental and Social Sustainability, University of Utah Health, Salt Lake City, UT, USA

## Introduction

The climate crisis has been called the greatest threat to health of the 21^st^ century. Healthcare accounts for 8.5% of total US greenhouse gas emissions,^1^ and the US healthcare system contributes more to global greenhouse gas emissions than any other healthcare system worldwide.^2^ Pharmaceuticals account for 10% of total healthcare emissions in the United States,^2^ with the carbon footprint across various stages of healthcare categorized into three scopes. Scope 3 includes all healthcare-related indirect emissions across fifteen categories, one of which is indirect emissions associated with the production and distribution of pharmaceuticals.^2^ Overall, pharmaceuticals account for approximately 18% of scope 3 emissions.^2^ One potential strategy to mitigate harmful emissions is to measure emissions encompassed in Scope 3 in the form of waste associated with medications lacking a clear indication.

30%–50% of antibiotic use across US healthcare is unnecessary.^3^ Antibiotic Stewardship encompasses efforts to measure and improve antibiotic prescribing with the goal of minimizing unintended consequences of unnecessary antibiotic prescribing. Antibiotic stewardship commonly focuses on development of diagnostic and treatment pathways, education, antibiotic restrictions, and provider feedback. Quantifying antibiotic-associated greenhouse gas emissions may be another tool for antibiotic stewardship to influence prescribing. We aimed to measure the greenhouse gas emissions from waste associated with unnecessary antibiotic prescriptions in the United States (US).

## Methods

We obtained sample waste from an outpatient antibiotic prescription from the University of Utah Outpatient Pharmacy including a paper bag, paper leaflet insert, and plastic prescription bottle. We calculated emissions for the waste based on the weight of each item using the US Environmental Protection Agency (EPA) greenhouse gas emission factors.^4^ Waste emissions are calculated by multiplying the total mass of each item by the appropriate emission factor depending on the waste type (eg, paper or plastic) and waste treatment method which we assumed to be landfilled.

We calculated unnecessary antibiotic prescriptions based on the estimated percent of unnecessary antibiotic prescriptions (28%) in 2014–2015 multiplied by the number of prescriptions in the US in 2022.^3,5^ Waste emissions of an individual prescription were multiplied by the number of unnecessary antibiotic prescriptions to arrive at a total landfilled waste emissions. We used the EPA’s Greenhouse Gas Equivalencies Calculator to convert emissions into concrete greenhouse gas equivalents that providers and patients can understand.^6^

## Results

An individual prescription has 32g of paper waste (paper bag 10g and leaflet 22g) and 15g of plastic bottle waste. Based on published estimates, there were 236,100,000 antibiotic prescriptions (Table 1) in the US in 2022, with 66,108,000 of them likely unnecessary.^3^ Our estimates suggest these prescriptions led to 1887.374 CO_2_e/ton of greenhouse gas emissions, the same as driving 4,838,375 miles by an average gasoline-powered vehicle, or 194.3 times around the equatorial circumference of the Earth.

**Table 1.** Estimates of waste emissions due to unnecessary outpatient antibiotic prescriptions in the US, 2014–2015

	Estimated mean no. of antibiotic prescriptions	Estimated no. of unnecessary antibiotic prescriptions	Paper weight^a^	Paper landfilled waste emissions (MT CO_2_ e/ton)^b^	Plastic weight^c^	Plastic landfilled waste emissions (MT CO_2_ e/ton)^d^	Total landfilled waste emissions (MT CO_2_ e/ton)	Greenhouse Gas Equivalent in miles driven by an average gasoline-powered vehicle
< 20 years of age	48,500,000	13,580,000	434,560 kg	383.216	203,700 kg	4.491	387.707	993,906
≥ 20 years of age	187,600,000	52,528,000	1,680,896 kg	1482.296	787,920 kg	17.371	1499.667	3,844,470
**Total**	**236,100,000**	**66,108,000**	**2,115,456 kg**	**1865.512**	**991,620 kg**	**21.862**	**1887.374**	**4,838,375**

a Weight of paper leaflet = 22g; weight of paper bag = 10 g.

b Emissions factor of 0.80.

c Weight of plastic bottle = 15g.

d Emissions factor of 0.02.

## Discussion

To our knowledge, this is the first evaluation of the impact of unnecessary antibiotics in humans on greenhouse gas emissions, and we estimated annual unnecessary outpatient antibiotic prescriptions contribute the same emissions as driving an average gas-powered car around the Earth 194.3 times.

Our analysis likely significantly underestimates emissions due to our ability to only estimate solid waste associated with antibiotic prescriptions; notably excluding emissions from upstream production and transportation of antibiotics that would be included in scope 3. We also did not account for patients who may refuse packaging or use paper and/or plastic recycling. However, even with these limitations, estimates of annual scope 3 outpatient antibiotic waste emissions are immense.

There are numerous examples of low-value care in medicine, defined by patient clinical outcomes and cost; however, when adding possible environmental impacts, we contend these practices become even less valuable and arguably detrimental to society. Traditional stewardship strategies have limited impact on certain providers and patients, and we believe incorporating data on environmental waste and contributions to greenhouse gas emissions from unnecessary antibiotics could only strengthen antibiotic stewardship efforts and inform public health policy.
